# Comparison of different techniques for the management of venous steno-occlusive lesions during placement of peripherally inserted central catheter

**DOI:** 10.1038/s41598-021-89780-6

**Published:** 2021-05-13

**Authors:** Woo Jin Yang, Danbee Kang, Ji Hoon Shin, Eun Ho Jang, Seung Yeon Noh, Suyoung Park, Hee Ho Chu, Jong Woo Kim

**Affiliations:** 1 Department of Radiology, Korea University Guro Hospital, Korea University College of Medicine, 148, Gurodong-ro, Guro-gu, Seoul, 08308 Republic of Korea; 2 Department of Clinical Research Design and Evaluation, SAIHST, Sungkyunkwan University, Seoul, Gangnam-gu, Republic of Korea; 3 Department of Radiology, Asan Medical Center, University of Ulsan College of Medicine, Olymphic-ro 43 gil 88, Songpa-Gu, Seoul, 05505 Republic of Korea; 4 Department of Radiology, Ulsan City Hospital, 1007, Saneop-ro, Buk-gu, Ulsan, 44238 Republic of Korea; 5 Department of Radiology, Kyung Hee University Hospital, College of Medicine, Kyung Hee University, 23, Kyungheedae-ro, Dongdaemun-gu, Seoul, 02447 Republic of Korea; 6 Department of Radiology, Gil Medical Center, Gachon University College of Medicine, 21, Namdong-daero 774 beon-gil, Namdong-gu, Incheon, 21565 Republic of Korea

**Keywords:** Medical research, Outcomes research

## Abstract

The purpose of this study is to investigate strategies for peripherally inserted central catheter (PICC) placement in patients with venous steno-occlusive lesion (VSOL). We performed a retrospective cohort study in adults with central or peripheral VSOL who underwent PICC placement procedures from January 2015 to December 2018. Four different strategies [selecting alternative pathway/over the wire (SAP/OTW), percutaneous transluminal angioplasty (PTA), re-puncture in ipsilateral arm (RIA), and catheter placement in the contralateral arm (CICA)] were analyzed and we compared the clinical outcomes by strategy and compared the strategy between central and peripheral VSOLs. During 4 years, 258 PICC procedures performed in patients with VSOLs, 100 PICC were included in the analysis. The overall technical success rate of initial attempt with SAP/OTW was 32.2%. As a second-line technique, PTA was most frequently used in both central (100%) and peripheral (68.2%) VSOL groups. The clinical success rates within 2 months of SAP/OTW, PTA, RIA, CICA were 55.2%, 43.2%, 14.3%, and 33.3%, respectively (*P* = 0.24). In conclusion, when the SAP/OTW failed, the PTA can be preferred as a second-line technique for both central and peripheral VSOLs. When guidewire passage fails, the operator could adopt the RIA or CICA technique as an alternative method.

## Introduction

The use of peripherally inserted central catheter (PICC) has increased rapidly in modern medical practice due to the ease of placement, perceived safety, and cost-effectiveness compared with other central venous catheters^1,2^. Although the technical success rate of PICCs has been reported to be 74–100%^3–6^, in patients with unsuspected asymptomatic venous steno-occlusive lesion (VSOL), there might be difficulties in performing the procedures regardless of whether the catheter is placed through the VSOL or through other venous access routes^7^.

Previous studies reported an unexpected central VSOL frequency of 2%^7,8^. Including peripheral VSOLs for which the incidence is not well known, it is not uncommon to encounter VSOL. However, the procedural policies of PICC placement in patients with VSOL are different at every institution due to the lack of clinical guidelines. Only a few studies indicated that the strategies were able to solve these problems, and those studies were only conducted in patients with central VSOL^7,8^.

When the initial attempt fails due to VSOL, re-accessing the other vein of the ipsilateral or contralateral arm may be a relatively simple alternative. However, considering that most of the patients undergoing PICC placement require long-term treatment or have poor peripheral veins, it is desirable to protect other veins for future venous access. In this respect, although technically more challenging, percutaneous transluminal angioplasty (PTA) can be a suitable second-line strategy. On the other hand, if the PTA affects negative effect on clinical outcomes of PICC placement, such as dwell time and complication, the advantage of protecting other veins could be overshadowed. As far as the authors know, previous studies did not compare the clinical outcomes of various strategies used for VSOL. Thus, this study aimed to describe the PICC placement strategy in patients with VSOL and to compare clinical outcomes by strategy and anatomical location. We hypothesized that the clinical outcomes of various strategies would be comparable. If our hypothesis is correct, it will provide a rationale for the PTA as the most preferred second-line strategy when the initial attempt fails due to VSOL.

## Results

### Study population

**Figure 1:**
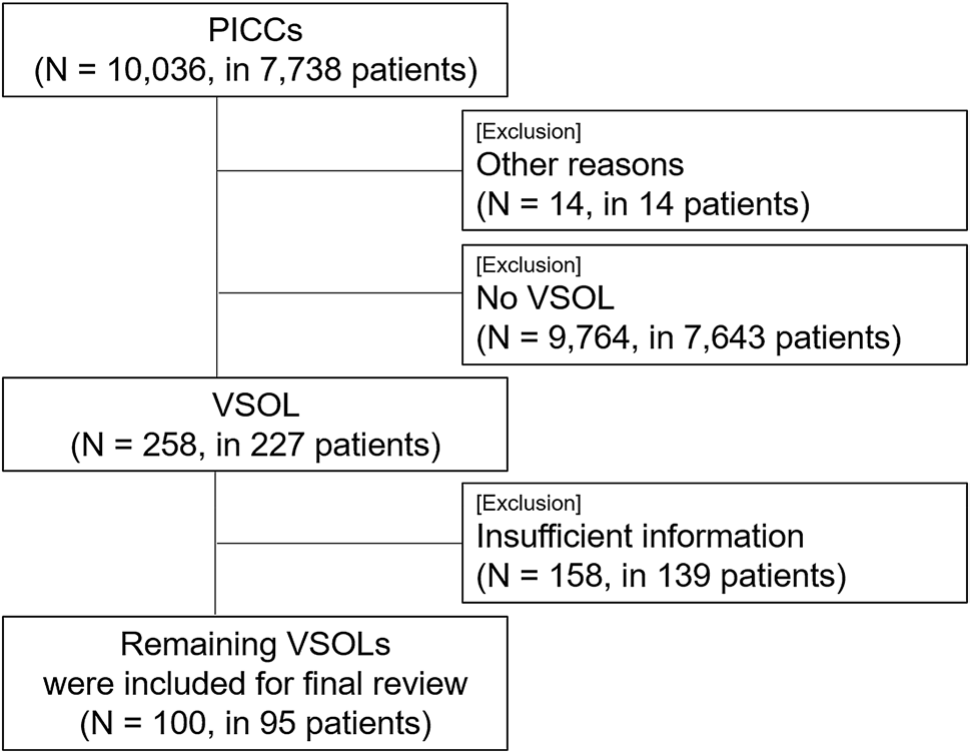
Flow diagram showing patient selection for analysis. *PICC* peripherally inserted central catheter, *VSOL* venous steno-occlusive lesion.

During the study period, 258 (2.6%, 258/10,036) PICC placement procedures were attempted in 227 patients who had unsuspected central or peripheral VSOL. After excluding 158 procedures in 139 patients who lacked the fundamental information, such as access vein or location, 100 PICC placement procedures performed in 95 patients were included in the final analysis to compare the technical and clinical outcomes (Fig. 1). Of the 100 procedures, the purpose of PICC placement, the patients’ major underlying and associated diseases necessitating PICC placement are described in Table 1 and Supplementary Table S1 & 2 online.

**Table 1:** Characteristics of the study participants (no. of procedures, N = 100).

Variables	Overall (N = 100)	Peripheral (N = 76)	Central (N = 24)	*P* values
Sex*				0.40
Male	45 (45.0)	36 (47.4)	9 (37.5)	
Female	55 (55.0)	40 (52.6)	15 (62.5)	
**Age**^‡^	62.6 (16.0)	62.0 (16.6)	64.4 (14.4)	0.52
Major underlying diseases*				
Diabetes mellitus	32 (32.0)	23 (30.3)	9 (37.5)	0.51
Hypertension	30 (30.0)	23 (30.3)	7 (29.2)	0.92
Ischemic heart disease	5 (5.0)	4 (5.3)	1 (4.2)	> 0.99
Cerebrovascular accident	6 (6.0)	4 (5.3)	2 (8.3)	0.63
Chronic kidney disease	14 (14.0)	9 (11.8)	5 (20.8)	0.27

*Data in parentheses are the percentages.

^‡^Data are expressed as mean (standard deviation).

### Characteristics of venous steno-occlusive lesion

In 95 patients (M:F = 43:52) with VSOL who underwent the procedure, central and peripheral VSOLs were detected in 24 (24%) and 76 (76%) patients, respectively (Table 1). In 24 patients, the central VSOLs detected were 5 occlusion and 19 stenosis, and distributed in the subclavian-brachiocephalic vein junction (n = 11), subclavian vein (n = 7), brachiocephalic vein (n = 3), axillary-subclavian vein junction (n = 1), brachiocephalic vein-superior vena cava (SVC) junction (n = 1), and SVC (n = 1). In 76 patients, the peripheral VSOLs detected were 6 occlusion and 70 stenosis, and were distributed in the basilic vein (n = 26), brachial vein (n = 20), axillary vein (n = 14), basilic-axillary vein junction (n = 9), brachial-axillary vein junction (n = 6), and cephalic vein (n = 1).

### Guidewire passage

Successful guidewire passage through the VSOL was achieved in 90 PICC procedures (90%, 90/100). The overall frequency of guidewire passage failure was 10% (n = 10), and there was no difference between central (8.3%, 2/24) and peripheral (10.5%, 8/76) VSOLs (*P* = 0.76).

**Figure 2:**
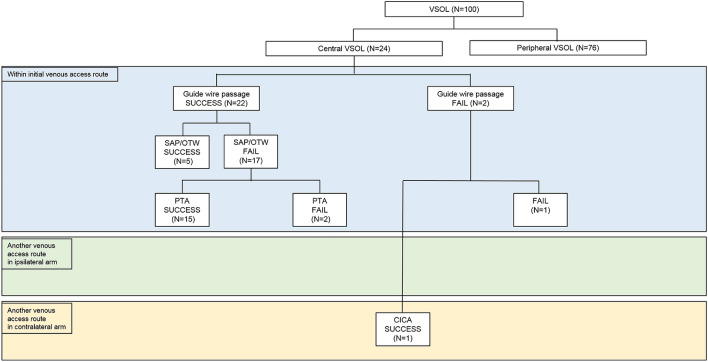
Flow diagram summarizing the various strategies used in patients with central venous steno-occlusive lesion. *VSOL* venous steno-occlusive lesion, *SAP/OTW* selecting alternative pathway/over the wire technique, *PTA* percutaneous transluminal angioplasty technique, *RIA* re-puncture in ipsilateral arm technique, *CICA* catheter placement in contralateral arm technique.

**Figure 3:**
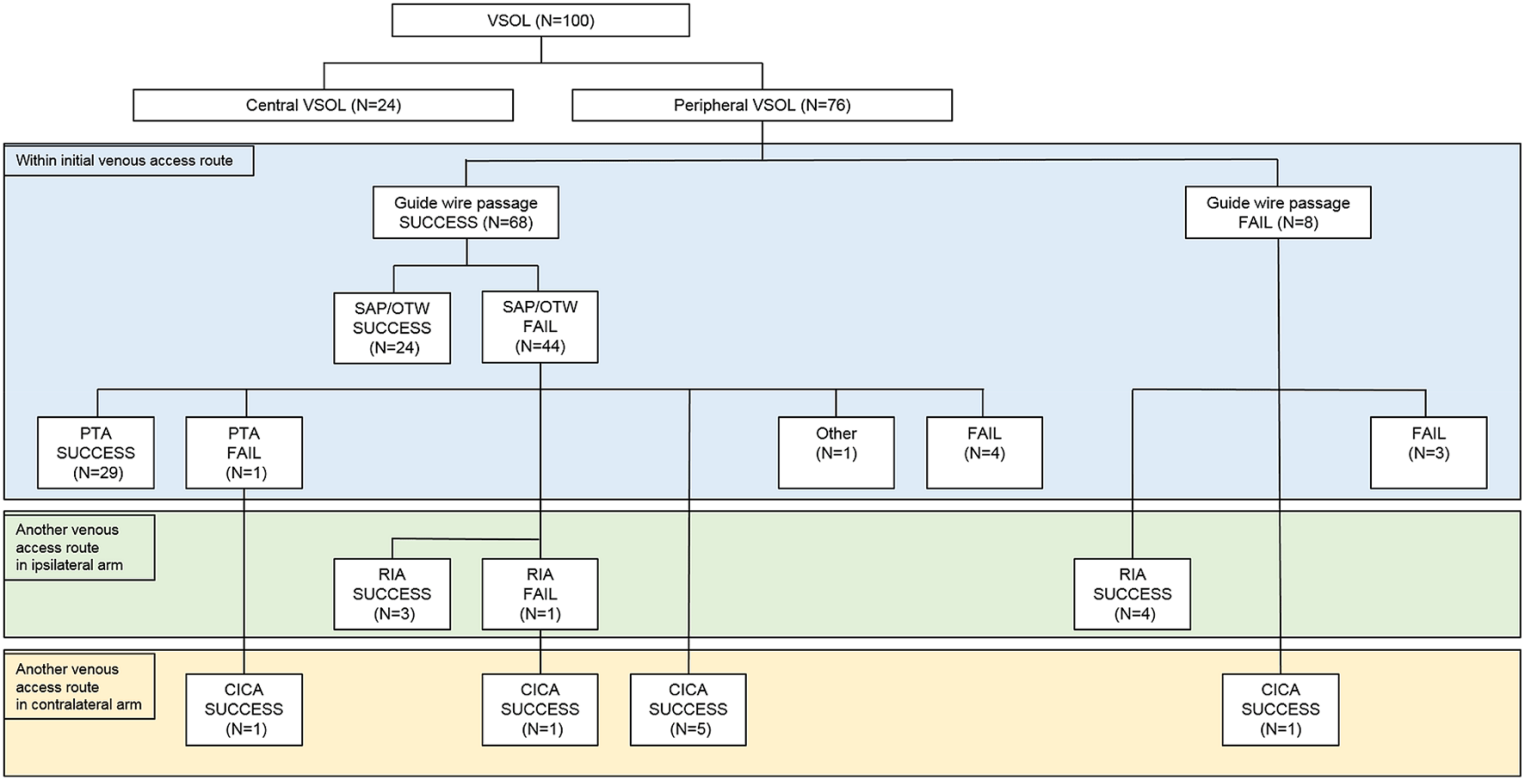
Flow diagram summarizing the various strategies used in patients with peripheral venous steno-occlusive lesion. *VSOL* venous steno-occlusive lesion, *SAP/OTW* selecting alternative pathway/over the wire technique, *PTA* percutaneous transluminal angioplasty technique, *RIA* re-puncture in ipsilateral arm technique, *CICA* catheter placement in contralateral arm technique.

In 10 guidewire passage failure procedures, re-puncture in the ipsilateral arm (RIA) or catheter placement in the contralateral arm (CICA) technique was utilized in four (all peripheral VSOLs) and two (one central and one peripheral VSOLs) procedures, respectively, while further interventions were no longer performed in the remaining four (one central and three peripheral VSOLs) procedures (Figs. 2 and 3).

### SAP/OTW (selecting alternative pathway/over the wire) technique

**Figure 4:**
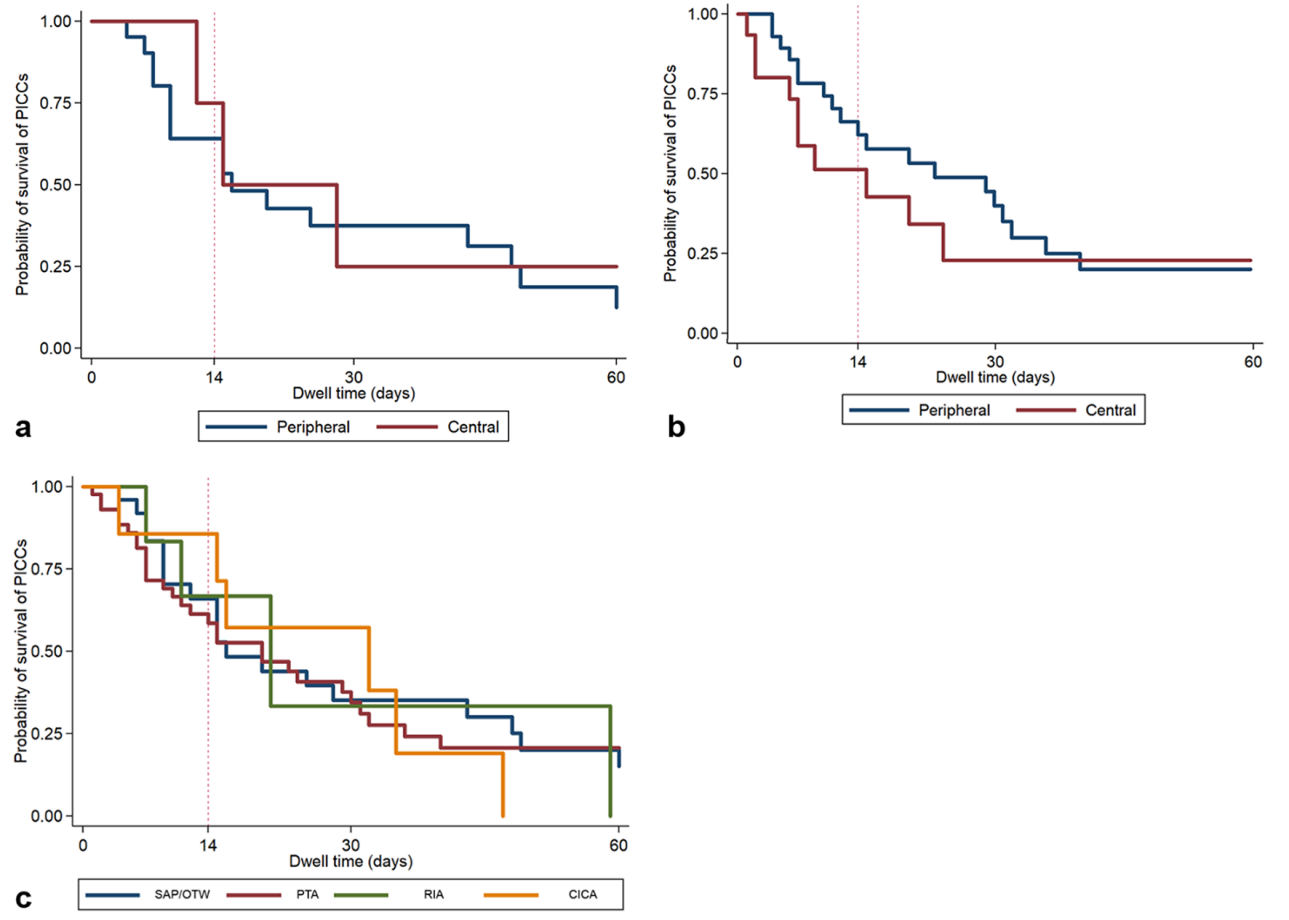
Kaplan–Meier estimate of dwell time of PICC placed, (**a**) using the SAP/OTW technique, (**b**) using PTA technique, and (**c**) by the last technique. The vertical dashed line depicts the time point of 14 days, while the end of x-axis represents the time point of 60 days. These time points are the two end points for assessing clinical success rate. *PICC* peripherally inserted central catheter, *SAP/OTW* selecting alternative pathway/over the wire technique, *PTA* percutaneous transluminal angioplasty technique, *RIA* re-puncture in ipsilateral arm technique, *CICA* catheter placement in contralateral arm technique.

In all cases with successful cannulation (90 procedures in 86 patients), the SAP/OTW technique was initially applied for catheter placement. The overall technical success rate of SAP/OTW technique was 32.2% (29/90). Although it was not significantly different, the peripheral VSOL group was more likely to succeed than the central VSOL group (35.3% (24/68) vs. 22.7% (5/22), *P* = 0.27) (Table 2). The clinical success rate within 2 weeks was not different between the central (80.0%, 4/5) and peripheral (75.0%, 18/24) VSOL groups (*P* = 0.81). The clinical success rate within 2 months was not also different between the central (60.0%, 3/5) and peripheral (54.2%, 13/24) VSOL groups (*P* = 0.60) (Table 2). The median dwell time was 15 and 12 days in the central and peripheral VSOL groups, respectively (*P* = 0.73) (Fig. 4a).

**Table 2:** Comparison of procedures used between central and peripheral VSOL groups (no. of procedures, n = 100).

Variables	Overall (N = 100)	Central (N = 24)	Peripheral (N = 76)	*P* values
**Guide wire passage, success**	90 (90.0)	22 (91.7)	68 (89.5)	0.76
**PICCs insert using SAP/OTW (N = 90)**				0.27
Yes	29 (32.2)	5 (22.7)	24 (35.3)	
Clinical success within 2 weeks	22 (75.9)	4 (80.0)	18 (75.0)	0.81
Clinical success within 2 months	16 (55.2)	3 (60.0)	13 (54.2)	0.60
Dwell time (days)*	15 (7–37)	15 (12–28)	12 (6.5–40)	0.73
Complications	5 (17.2)	0	5 (20.8)	0.36
Infection	5 (17.2)	0	5 (20.8)	0.36
No	61 (67.8)	17 (77.3)	44 (64.4)	
**Second-line methods after failure of SAP/OTW (N = 61)**				0.15
PTA	47 (77.0)	17 (100)	30 (68.2)	
RIA	4 (6.6)	0	4 (9.1)	
CICA	5 (8.2)	0	5 (11.4)	
Others	1 (1.6)	0	1 (2.3)	
Fail, without a 2nd attempt	4 (6.6)	0	4 (9.1)	
**Third-line methods after failure of 2nd line methods (N = 2)**				
CICA	2 (100)	0	2 (100)	–

Data in parentheses are the percentages except for the dwell time.

*Calculated using the Kaplan–Meier Methods. Data in parentheses are the ranges.

### PTA (percutaneous transluminal angioplasty) technique

As a second-line technique after the first attempt of SAP/OTW technique failed, PTA technique was most frequently used in both the central and peripheral VSOL groups (100% vs. 68.2%). The overall technical success rate of the PTA technique was 93.6% (44/47). Although it was not significantly different, the peripheral VSOL group was more likely to succeed than the central VSOL group (96.7% (29/30) vs. 88.2% (15/17), *P* = 0.29). The clinical success rate within 2 weeks and 2 months were higher in the peripheral VSOL group (2 weeks = 75.9%, 2 months = 44.8%) than in the central VSOL group (2 weeks = 46.7%, 2 months = 40.0%), but it was not statically significant. The median dwell time was also longer in the peripheral VSOL group than in the central VSOL group (14 days vs. 9 days, *P* = 0.65) (Table 3, Fig. 4b).

**Table 3:** Summary of technical and clinical outcomes of PICC placed using the PTA technique.

Parameter	Overall (N = 47)	Central (N = 17)	Peripheral (N = 30)	*P* values
**Technical success**				0.29
Yes	44 (93.6)	15 (88.2)	29 (96.7)	
Clinical success within 2 weeks	29 (65.9)	7 (46.7)	22 (75.9)	0.06
Clinical success within 2 months	19 (43.2)	6 (40.0)	13 (44.8)	0.51
Dwell time (days)*	13.5 (6.5–30)	9 (6–23)	14 (7–31)	0.65
No	3 (6.4)	2 (11.8)	1 (3.3)	

Data in parentheses are the percentages except for the dwell time.

*Calculated using the Kaplan–Meier Methods. Data in parentheses are the ranges.

### Clinical outcome according to the last technique

The technical success rates were 32.2%, 93.6%, 87.5%, and 100% in SAP/OTW, PTA, RIA, and CICA groups, respectively. Among the technical success procedures, the clinical success rate within 2 weeks of SAP/OTW, PTA, RIA, and CICA groups were 75.9%, 65.9%, 71.4%, and 77.8%, respectively (*P* = 0.84). The clinical success rate within 2 months were 55.2%, 43.2%, 14.3%, and 33.3% in SAP/OTW, PTA, RIA, and CICA groups, respectively (*P* = 0.24). The median dwell time was 15, 13.5, 16, and 16 days in SAP/OTW, PTA, RIA, and CICA groups, respectively (*P* = 0.99) (Table 4 and Fig. 4c).

**Table 4:** Summary of the clinical outcomes according to the last technique (no. of procedures, n = 89).

Parameter	SAP/OTW (N = 29)	PTA (N = 44)	RIA (N = 7)	CICA (N = 9)	P values
**Clinical success within 2 weeks**	22 (75.9)	29 (65.9)	5 (71.4)	7 (77.8)	0.84
**Clinical success within 2 months**	16 (55.2)	19 (43.2)	1 (14.3)	3 (33.3)	0.24
Dwell time (days)*	15 (7–37)	13.5 (6.5–30)	16 (7–21)	16 (4–32)	0.99
**Complications**					
Vein injury	2 (6.9)	4 (9.1)	1 (14.3)	0	0.73
Infection	5 (17.2)	10 (22.7)	1 (14.3)	1 (11.1)	0.88
Edema	0	2 (4.6)	6 (85.7)	0	0.71

Data in parentheses are the percentages except for the dwell time.

*Calculated using the Kaplan–Meier Methods. Data in parentheses are the ranges.

On comparing the complication rate, the incidence of vein injury was found to be highest in the RIA group (14.3%, 1/7) followed by the PTA (9.1%, 4/44) and SAP/OTW groups (6.9%, 2/29) and all of them occurred in the peripheral VSOLs. The incidence of infection was highest in the PTA group (22.7%, 10/44) followed by the SAP/OTW, RIA, and CICA groups. The incidence of edema was also highest in the RIA group (85.7%, 6/7). Each complication was not significantly different among the four groups (Table 4).

### Analysis of technical options

Ipsilateral technical success rate was achieved in 83.3% (20/24) and 80.3% (61/76) of the procedures performed in patients with central and peripheral VSOLs, respectively (Figs. 2 and 3).

When guidewire passage was successful, SAP/OTW technique was attempted in all central and peripheral VSOLs. In central VSOLs, when the SAP/OTW technique failed, the PTA technique was attempted with a high success rate (88.2%, 15/17). On the contrary, in peripheral VSOLs, when the SAP/OTW technique failed, many technical options were utilized: the PTA (68.2%, 30/44), RIA (9.1%, 4/44), CICA (11.4%, 5/44), and peel-away sheath dilation techniques (2.7%, 1/44, marked as “Other” in Fig. 3). Meanwhile, no further procedure was attempted in 9.1% (4/44) of the patients. The peel-away sheath dilation technique involved the dilation of the VSOL using a peel-away sheath as the lesion was located in the peripheral area and close to the puncture site.

When the guidewire passage failed, the SAP/OTW or PTA techniques could not be used. In central VSOLs (n = 2), the RIA technique could not be applied; therefore, the CICA technique was utilized in one patient. In peripheral VSOLs, either RIA (50%, 4/8) or CICA (12.5%, 1/8) technique was utilized, while no further attempt was made in three patients.

## Discussion

In this study, the overall technical success rate of SAP/OTW technique was 32.2% (29/90). When the initial attempt with SAP/OTW failed, PTA technique was most frequently used in both central and peripheral VSOL groups. The clinical outcomes of SAP/OTW and PTA techniques, such as clinical success rate, dwell time, and complication rate, were not significantly different between the central and peripheral VSOL groups. We also found that the overall clinical outcomes of SAP/OTW, PTA, RIA, and CICA techniques were not significantly different.

Although the success rate of SAP/OTW technique was not high (32.2%), it was a basic technique in patients with VSOL and initially applied in all cases with successful guidewire passage. This study showed that the SAP/OTW technique had a higher technical success rate in peripheral VSOLs than in central VSOLs, although this was not statistically significant. It seems secondary to anatomical network between the peripheral veins and the relatively close location of the VSOL from the pushing point.

The PTA technique is a well-recognized treatment for central or peripheral VSOL^9–11^. However, most previous studies evaluated vascular access in a hemodialysis population^12–14^, and only a few studies investigated the efficacy of the PTA technique in patients requiring PICC placement^7,8^. In our study, the PTA technique was applied in 77.0% of patients who failed PICC placement using the SAP/OTW technique, and the overall technical success rate of PTA technique was very high (93.6%). Because of this, we could markedly improve the success rate of PICC placement within the initial venous access route. Therefore, the PTA technique can be considered helpful in preserving other potentially usable veins. Furthermore, clinical outcomes, such as clinical success rate, dwell time, and complication rate, were not significantly different among the four groups (SAP/OTW, PTA, RIA, and CICA groups). Considering that the PICCs of PTA group were placed in the vein with the most severe VSOL, these results are encouraging and advocate more assertive and frequent application of the PTA technique in patients with VSOL. Thus, the PTA technique can be preferred as a second-line technique in patients with VSOL.

To date, there have been no studies on how to perform PICC placement in patients with peripheral VSOL. In the present study, the clinical outcomes of SAP/OTW and PTA techniques were not significantly different between the central and peripheral VSOL groups. Thus, SAP/OTW and PTA techniques can also be preferred for peripheral VSOL as well as for central VSOL. However, if guidewire passage fails, PICC cannot be introduced until another venous route is secured because SAP/OTW or PTA techniques cannot be applied. In this situation, the operator could adopt the RIA or CICA technique as a second-line method.

This study has several limitations. First, this study was a retrospective study, and there was a considerable loss of cases with VSOLs due to insufficient data. Second, PICCs of variable size and types were used in this study. The catheter factors may have considerable effect on the technical and clinical outcomes. Third, analysis for the length and classification (whether stenosis or occlusion) of VSOL was not performed. Because of the nature of the retrograde image review, the accuracy of the evaluation was limited. Finally, this is a single-center study and outcomes from our clinical practice may differ from other centers. External validation is needed from different centers.

Despite several limitations, this study has values as evidence suggesting a clinical guideline for PICC placement in patients with VSOL. In conclusion, when the SAP/OTW technique failed, the PTA can be preferred as a second-line technique for both central and peripheral VSOLs. When guidewire passage fails, the operator could adopt the RIA or CICA technique as an alternative method.

## Materials and methods

### Participants

The institutional review board of author's institute (“Asan Medical Center institutional review board”) approved this retrospective study, and the requirement for written informed consent was waived. All methods were carried out in accordance with relevant guidelines and regulations.

This retrospective cohort study was conducted in a tertiary hospital from January 2015 to December 2018. During the study period, 10,036 PICC placement procedures were attempted in 7738 adult patients (aged 18 years and older) and the images and medical records of these procedures were retrospectively reviewed. Among this population, we excluded procedures performed in patients without VSOL and patients who experienced difficulty for reasons other than VSOL (Fig. 1).

### PICC procedures

All PICCs were placed in an interventional radiology suite under ultrasound and fluoroscopic guidance by more than 10 operators. Four types of PICCs made by three manufacturers were used: TURBO-JECT POWER-INJECTABLE PICC (COOK, Inc., Bloomington, Indiana; double lumen, 5F), XCELA POWER INJECTABLE PICC (NAVILYST MEDICAL, Marlborough, Massachusetts; double lumen, 5F), PRO-PICC CT INJECTABLE CATHETERS (MEDCOMP, Harleysville, Pennsylvania; double lumen, 5F), and PRO-PICC CT INJECTABLE CATHETERS (MEDCOMP, Harleysville, Pennsylvania; Triple lumen, 6F). The catheters were generally placed in the right arm via the basilic, brachial, or cephalic veins in decreasing order of preference. The left arm was used only if the right arm was inaccessible or the attempt on the right arm failed.

The peripheral arm vein was punctured with a 21-gauge micropuncture needle under ultrasound guidance. Once the 0.018-inch guidewire had been introduced into the right atrium without resistance, a peel-away sheath was inserted through the guidewire. After measuring the actual length from the puncture site to the right atrium using the 0.018-inch guidewire, the catheter was cut adequately and the PICC was advanced to the right atrium. If an operator felt any resistance during passage of the guidewire or the PICC catheter, venography was performed using a peel-away sheath or a catheter introduced through the arm veins.

If venography revealed VSOL in the central or peripheral veins, the available technical options were as follows: (1) selecting alternative pathway/over the wire (SAP/OTW) technique, (2) percutaneous transluminal angioplasty (PTA) technique, (3) re-puncture in ipsilateral arm (RIA) technique, and (4) catheter placement in contralateral arm (CICA) technique.

The SAP/OTW technique was utilized during the selection of more patent veins with an 0.018-inch guidewire (SAP technique) and/or when the catheter was inserted over the 0.018-inch guidewire through the VSOL with a gentle, forceful push (OTW technique). In some cases, an additional 0.018-inch guidewire was inserted through another PICC lumen for more robust support. Since the SAP and OTW techniques were applied simultaneously in real daily practice, it was impossible to classify these into two distinct groups. Therefore, all cases with successful guidewire passage could be classified into one group, namely, SAP/OTW technique.

The PTA technique could be considered an alternative option when the SAP/OTW technique was impossible to perform. Even if the PTA technique was unsuccessful, the initial access route was abandoned, and the RIA or CICA technique was utilized to perform PICC placement through the new access route.

The decision regarding which of the above options should have been chosen with priority depended entirely on the operator’s choice.

### Assessment of study outcomes

Medical records and radiology reports of patients who underwent PICC placement were reviewed. VSOL was classified into stenosis or occlusion; stenosis was defined as luminal narrowing of a vessel compared with the adjacent normal segment, while occlusion was defined as non-opacified vessel on the venography image. The strategy utilized for PICC placement was analyzed in patients with VSOL.

The additional information obtained included patient characteristics such as diagnosis, indication for PICC placement, reasons for PICC removal, and PICC-related complications.

### Definitions

Technical success was defined as successful placement of a catheter tip between the low superior vena cava and the upper right atrium. Clinical success within 2 weeks was defined as patent catheterization for more than 2 weeks or removal of catheter within 2 weeks as the treatment had already been completed. Clinical success within 2 months was defined as patent catheterization for more than 2 months or removal of catheter within 2 months as the treatment had already been completed. PICC dwell time was calculated as the interval between the date of placement and that of removal. Vein injury was defined as extravasation of contrast material into the soft tissues accompanied by vessel wall irregularity. Catheter malfunction was defined as failure to withdraw blood or flush the line. Infection was determined by clinical signs, including fever, purulent discharge from the placement site, or evidence of phlebitis not responding to conservative management.

### Statistical analysis

Descriptive analysis was performed. The characteristics, technical and clinical success rates, and complication rate of central and peripheral VSOL groups were compared using t-test and chi-square test or Fisher’s exact test for continuous variables and categorical variables, respectively. The clinical success and complication rates, according the last technique, were also compared in the same manner. However, regarding the technical success rates of the last technique, there was significant heterogeneity of technical strategies among the procedures performed in the included patients; therefore, descriptive analysis was only performed. The patency curves for PICCs were estimated in accordance with the Kaplan–Meier method.

All analyses were performed using STATA version 14 (StataCorp LP, College Station, TX, USA). Differences of *P* < 0.05 were considered significant.

## Supplementary information

Supplementary Information.

## Data Availability

The datasets generated during and/or analyzed during the current study are not publicly available due to concerns about backtracking of personal information of study subjects but are available from the corresponding author on reasonable request.
