# Author Correction: Comparison of different techniques for the management of venous steno-occlusive lesions during placement of peripherally inserted central catheter

**DOI:** 10.1038/s41598-021-96542-x

**Published:** 2021-08-25

**Authors:** Woo Jin Yang, Danbee Kang, Ji Hoon Shin, Eun Ho Jang, Seung Yeon Noh, Suyoung Park, Hee Ho Chu, Jong Woo Kim

**Affiliations:** 1 Department of Radiology, Korea University Guro Hospital, Korea University College of Medicine, 148, Gurodong-ro, Guro-gu, Seoul, 08308 Republic of Korea; 2 Department of Clinical Research Design and Evaluation, SAIHST, Sungkyunkwan University, Gangnam-gu, Seoul, Republic of Korea; 3 Department of Radiology, Asan Medical Center, University of Ulsan College of Medicine, Olymphic-ro 43 gil 88, Songpa-Gu, Seoul, 05505 Republic of Korea; 4 Department of Radiology, Ulsan City Hospital, 1007, Saneop-ro, Buk-gu, Ulsan, 44238 Republic of Korea; 5 Department of Radiology, Kyung Hee University Hospital, College of Medicine, Kyung Hee University, 23, Kyungheedae-ro, Dongdaemun-gu, Seoul, 02447 Republic of Korea; 6 Department of Radiology, Gil Medical Center, Gachon University College of Medicine, 21, Namdong-daero 774 beon-gil, Namdong-gu, Incheon, 21565 Republic of Korea

Correction to: *Scientific Reports* 10.1038/s41598-021-89780-6, published online 13 May 2021

The original version of this Article contained errors in the Affiliations.

Woo Jin Yang was incorrectly affiliated with “Department of Medicine, Graduate School, Kyung Hee University, 26, Kyungheedae-ro, Dongdaemun-gu, Seoul, 02447, Republic of Korea”. The correct affiliation is listed below.

Department of Radiology, Korea University Guro Hospital, Korea University College of Medicine, 148, Gurodong-ro, Guro-gu, Seoul, 08308, Republic of Korea.

Ji Hoon Shin was incorrectly affiliated with “Department of Radiology, College of Medicine Asan Medical Center, University of Ulsan, 86, Asanbyeongwon-gil, Songpa-gu, Seoul, 138-736, Korea”. The correct affiliation is listed below.

Department of Radiology, Asan Medical Center, University of Ulsan College of Medicine, Olymphic-ro 43 gil 88, Songpa-Gu, Seoul, 05505, Republic of Korea.

As a result of the changes, the Affiliations have been renumbered.

The original Article and the accompanying Supplementary Information file have been corrected.

