# Supplementary material for: Comparison of different techniques for the management of venous steno-occlusive lesions during placement of peripherally inserted central catheter
Source: Sci Rep. 2021 May 13;11:10234. doi: 10.1038/s41598-021-89780-6 (PMC8119704; doi:10.1038/s41598-021-89780-6)
Supplement: Supplementary file 1 — Supplementary Information. [file 41598_2021_89780_MOESM1_ESM.docx]

**Comparison of different techniques for the management of venous steno-occlusive lesions during placement of peripherally inserted central catheter**

Woo Jin Yang, MD^1†^; Danbee Kang, PhD^2†^; Ji Hoon Shin, MD, PhD^3*^; Eun Ho Jang, MD^4^; Seung Yeon Noh, MD^5^; Suyoung Park, MD^6^; Hee Ho Chu, MD^3^; and Jong Woo Kim, MD^3^

^1^Department of Radiology, Korea University Guro Hospital, Korea University College of Medicine, 148, Gurodong-ro, Guro-gu, Seoul 08308, Republic of Korea

^2^Department of Clinical Research Design and Evaluation, SAIHST, Sungkyunkwan University, Seoul, Gangnam-gu, Republic of Korea

^3^Department of Radiology, Asan Medical Center, University of Ulsan College of Medicine, Olymphic-ro 43 gil 88, Songpa-Gu, Seoul 05505, Republic of Korea

^4^Department of Radiology, Ulsan City Hospital, 1007, Saneop-ro, Buk-gu, Ulsan, 44238, Republic of Korea

^5^Department of Radiology, Kyung Hee University Hospital, College of Medicine, Kyung Hee University, 23, Kyungheedae-ro, Dongdaemun-gu, Seoul, 02447, Republic of Korea

^6^Department of Radiology, Gil Medical Center, Gachon University College of Medicine, 21, Namdong-daero 774 beon-gil, Namdong-gu, Incheon 21565, Republic of Korea

†These authors contributed equally to this work and are co-first authors.

***Corresponding author**

Ji Hoon Shin, MD

Department of Radiology, Asan Medical Center, University of Ulsan College of Medicine, Olymphic-ro 43 gil 88, Songpa-Gu, Seoul 05505, Republic of Korea

Tel: 82-2-3010-4380

Fax: 82-2-476-0090

E-mail: jhshin@amc.seoul.kr


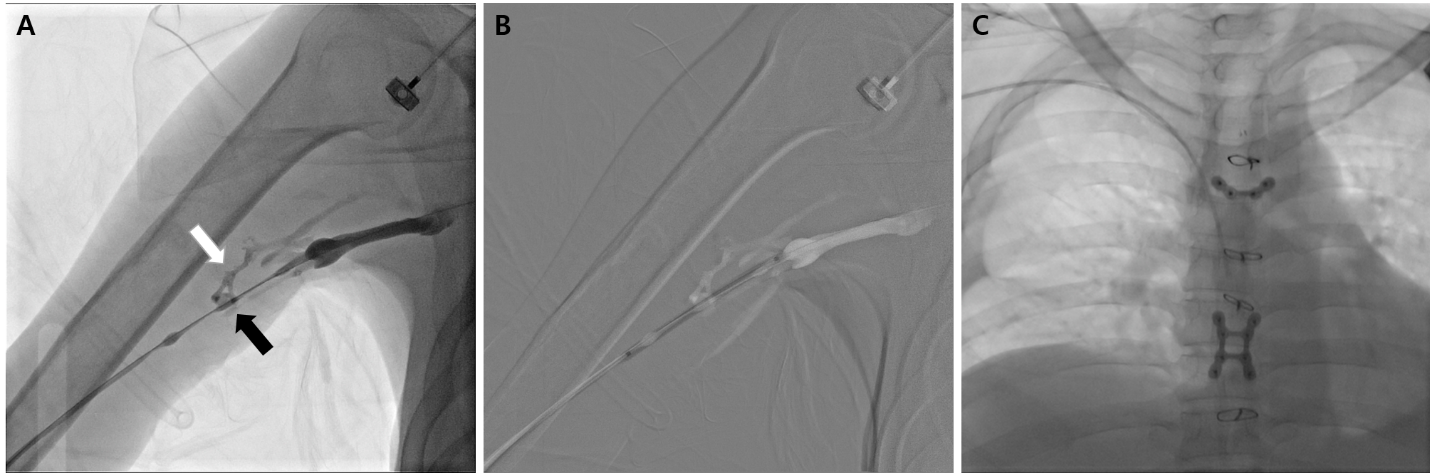


Supplementary Figure S1. Example of peripheral VSOL. Images of a 40-year-old man with peritonitis who was referred for PICC placement for total parenteral nutrition. (A) Venography image shows severe segmental stenosis of the right brachial vein (black arrow) with the development of collateral veins (white arrow). (B) Balloon angioplasty was performed using a 4×60 mm balloon catheter. (C) The PICC catheter was successfully placed. (All figures in supplementary information courtesy of Asan Medical Center)


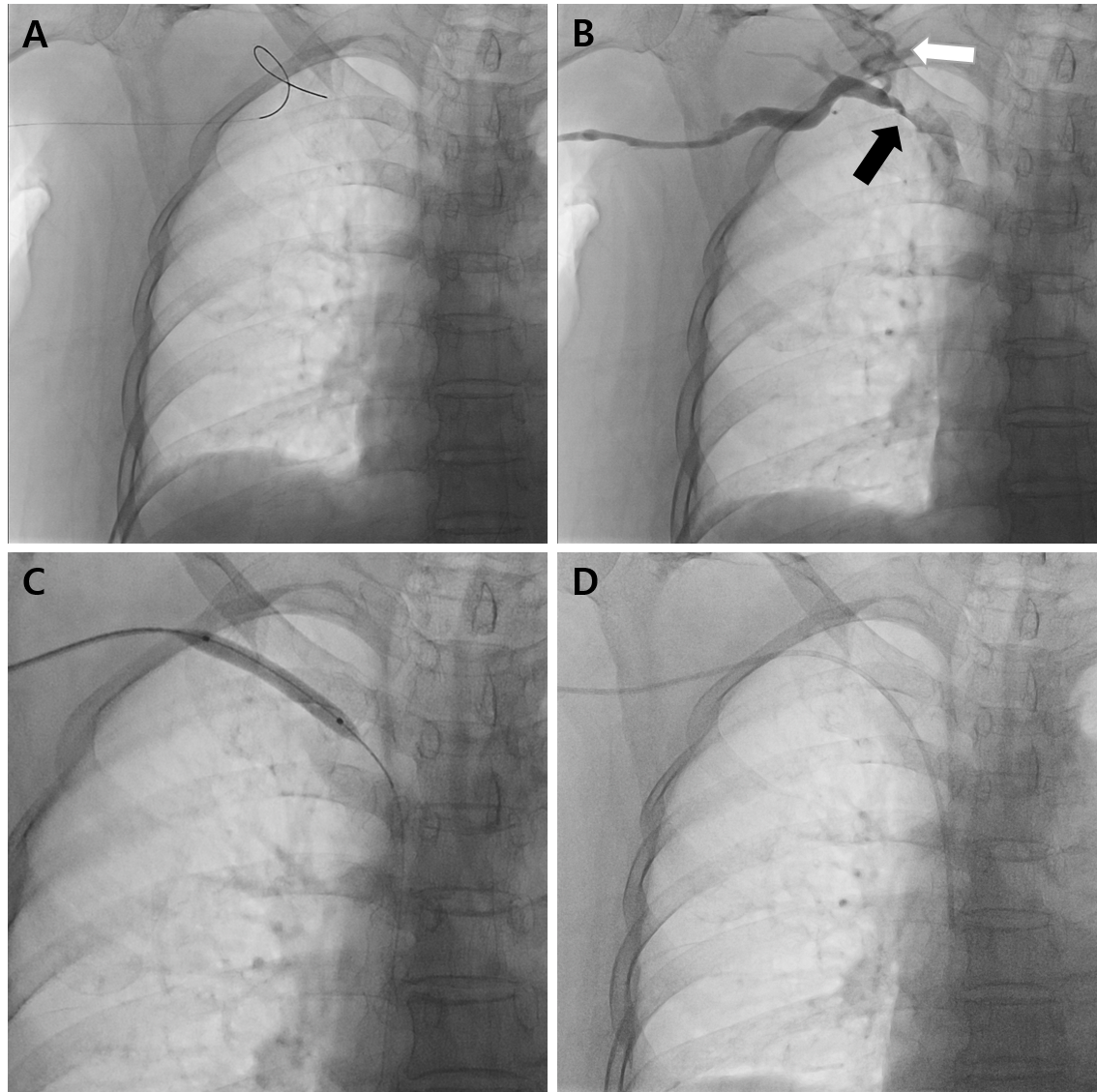


Supplementary Figure S2. Example of central VSOL. A 78-year-old woman with pneumonia who was referred for PICC placement for intravenous drug therapy. (A, B) Owing to resistance during guidewire advancement, a venography was performed, which shows severe focal stenosis in the right subclavian vein (black arrow) with the development of collateral veins (white arrow). (C, D) After balloon angioplasty using an 8×40 mm balloon catheter, the PICC catheter was placed successfully. (All figures in supplementary information courtesy of Asan Medical Center)

**Supplementary Table S1. Purpose of PICC placement in patients with VSOL (only determined in 55 procedures)**

| **Purpose** | **No. of procedures (N = 55)** | |
| --- | --- | --- |
| Intravenous drug therapy | | 36 (65%) |
| Total parenteral nutrition | | 14 (25%) |
| Chemotherapy | | 2 (4%) |
| Transfusion | | 2 (4%) |
| Hydration | | 1 (2%) |

Note. PICC = peripherally inserted central catheter.

**Supplementary Table S2. Associated diseases necessitating PICC placement in patients with VSOL**

| **Diseases** | **No. of procedures (N = 100)** | |
| --- | --- | --- |
| Infectious disease | | 32 (32%) |
| Malignant neoplasm | | 27 (27%) |
| Liver cirrhosis | | 6 (6%) |
| Status after organ transplantation | | 5 (5%) |
| Ileus | | 4 (4%) |
| Pancreatitis | | 4 (4%) |
| Hematologic disorder | | 3 (3%) |
| Cerebrovascular accident | | 3 (3%) |
| Inflammatory bowel disease | | 2 (%) |
| Bowel perforation | | 2 (2%) |
| Cardiomyopathy | | 2 (2%) |
| Heart failure | | 2 (2%) |
| Others | |  |
| Diabetes mellitus foot | | 1 (1%) |
| Acute myocardial infarction | | 1 (1%) |
| Pulmonary embolism | | 1 (1%) |
| Metabolic encephalopathy | | 1 (1%) |
| Ischemic colitis | | 1 (1%) |
| Cholecystitis | | 1 (1%) |
| Seizure | | 1 (1%) |
| Sialadenitis | | 1 (1%) |

Note. PICC = peripherally inserted central catheter.
